# Molecular epidemiology of *Giardia* spp. in northern Vietnam: Potential transmission between animals and humans

**DOI:** 10.1016/j.parepi.2020.e00193

**Published:** 2020-12-24

**Authors:** Hanako Iwashita, Tetsuhiro Sugamoto, Taichiro Takemura, Asako Tokizawa, Thiem Dinh Vu, Tuan Hai Nguyen, Tho Duc Pham, Na Ly Tran, Hang Thi Doan, Anh Hong Quynh Pham, Tetsu Yamashiro

**Affiliations:** a Department of International Affairs and Tropical Medicine, Tokyo Women's Medical University, 8-1 Kawada-cho, Shinjuku-ku, Tokyo 162-8666, Japan; b International Programs, Japan Anti-Tuberculosis Association, 3-1-24 Matsuyama, Kiyose-shi, Tokyo 204-8533, Japan; c Vietnam Research Station, Center for Infectious Disease Research in Asia and Africa, Institute of Tropical Medicine, Nagasaki University, 1-12-4 Sakamoto, Nagasaki-shi, Nagasaki 852-8523, Japan; d Research Center for Child Mental Development, Hamamatsu University School of Medicine, 1-20-1 Handayama, Higashi-ku, Hamamatsu city, Shizuoka 431-3192, Japan; e National Institute of Hygiene and Epidemiology, No.1 Yersin Street, Hai Ba Trung District, Hanoi 10000, Viet Nam; f International hospital Vinmec Times City, 458 Minh Khai, Vinh Tuy, Hai Ba Trung, Ha Noi, Viet Nam; g Division of Bio-Medical Science & Technology, Korea University of Science and Technology (UST), Daejeon 34113, Republic of Korea; h Department of Bacteriology, Graduate School of Medicine, University of the Ryukyus, 207 Uehara, Nishiharacho, Okinawa 903-0215, Japan

**Keywords:** *Giardia* spp., Zoonotic transmission, Zooanthroponotic transmission, Assemblage A, Assemblage E, Northern Vietnam, DFA, Direct Immunofluorescence Assay, *bg*, *beta-giardin*, *gdh*, *glutamate dehydrogenase*, *tpi*, *triose phosphate isomerase*

## Abstract

*Giardia* spp. is detected frequently in humans and animals. Although many studies have been conducted on the epidemiology of giardiasis, there is a scarcity of information on the genetic diversity and the dynamics of transmission of *Giardia* spp. in Vietnam. The zoonotic potential of *Giardia* spp. remains elusive. The objective of this study was to determine the genetic diversity of *Giardia* spp. in both humans and livestock to assess the existence of a route of infection between livestock and humans. Our goal was to assess the role animals play in the epidemiology of human infection in northern Vietnam. In Hien Khanh commune in northern Vietnam, 311 households with 1508 residents were randomly selected for a diarrheal cohort study. Of these, 2120 human diarrheal samples were collected from 1508 residents in 2014 and 2017. Of these, non-diarrheal samples were cross-sectionally collected from 471 residents. At the same site, livestock samples from buffalo, dairy and beef cattle, pigs, and dogs were collected. All stool samples were examined for *Giardia* spp. by Direct Immunofluorescence Assay (DFA) using fluorescent microscope. DNA extraction, PCR analysis of the 3 genes (*bg, gdh, tpi*), and sequencing analysis were continuously carried out. A total of 23 animal stool samples, 8 human non-diarrheal samples, and 36 human diarrheal samples were *Giardia* spp. were positive by PCR using the *bg* and *gdh* genes. *Giardia* spp. assemblage AII and E were detected in both animal samples and human samples in this study site. The detection of assemblage E in human stool samples suggests the first human case report in Vietnam. We assume that the unexpected human infection of all *Giardia* assemblages including A, B, and E may be due to an environment contaminated with animal and human feces in this village.

## Highlights

• *Giardia* spp. assemblage A and E have been detected in buffalo, cattle and pigs, as well as in humans, while assemblage B appeared only in humans.
• This was the first report of assemblage E in humans in Vietnam.
• Zoonotic transmission of assemblage AII and E could not be excluded in our study site.
• In addition to the ‘animal-to-human’ route, there potentially might be transmission routes from ‘humans-to-humans’ and/or ‘humans-to-animals’ in this study site.
• The novel genotype belonging to assemblage AIII has been isolated only in buffalo.

## Graphical abstract

**Figure:**
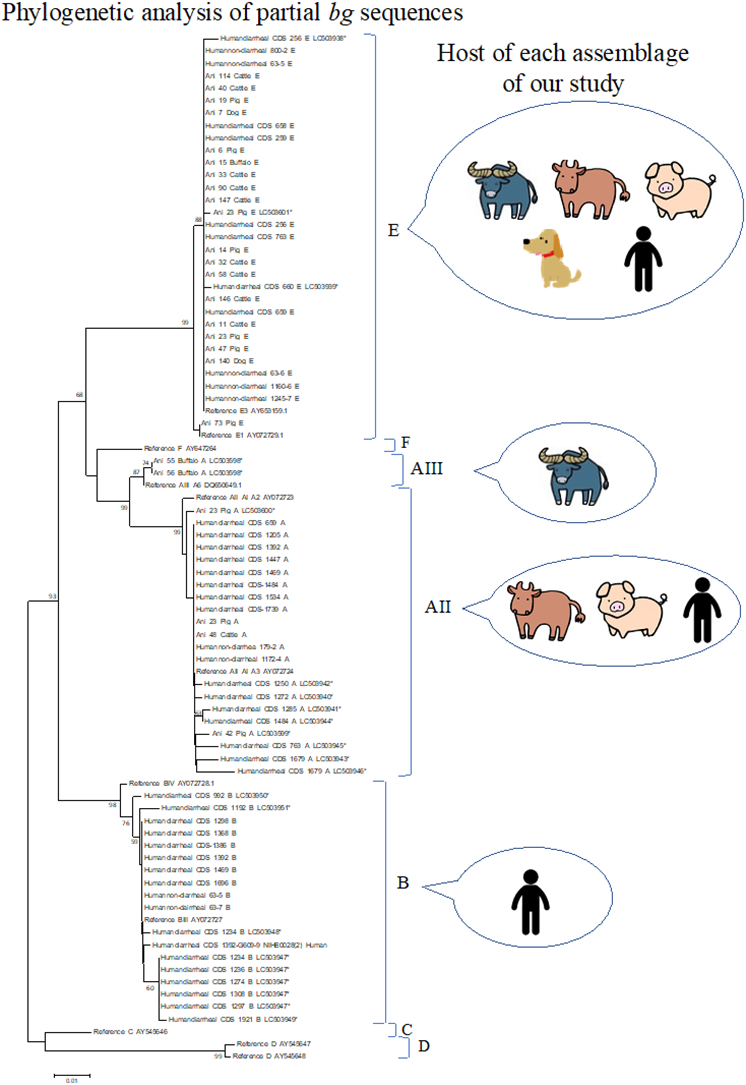

## 1 Background

*Giardia* spp.is the etiologic agent of giardiasis in humans and other animals throughout the world (Ryan and Cacciò, 2013). Previous epidemiological studies have shown that *Giardia* spp. is common in livestock worldwide. Similarly, in central Vietnam, Nguyen et al. detected *Giardia* spp. in cattle in small-scale farms (Nguyen et al., 2016). Researchers considered cattle, particularly pre-weaned calves, to be a major contributor to zoonotic infections (Abeywardena et al., 2012; Santín et al., 2009). In Vietnam, the raising of livestock including cattle and other animals is a traditional agricultural practice (Nguyen et al., 2016). The zoonotic transmission potential of *Giardia* spp. remains a major and unresolved issue.

*Giardia* spp. is divided into 8 morphologically identical genotypes or assemblages (A to H) (Ryan and Cacciò, 2013). These assemblages differ in their genetic characterization and host specificity is different (Feng and Xiao, 2011; Heyworth, 2016). Assemblage A and B tend to be detected in both humans and animals, while the remaining 6 assemblages (C to H) tend to be specific to animals (Cacciò et al., 2018; Ryan and Cacciò, 2013; Thompson and Monis, 2012). Although we could simply focus on the reports of human giardiasis caused by assemblages A and B, which are believed to have the potential for zoonotic transmission (Cacciò and Ryan, 2008; Cacciò et al., 2018), we cannot ignore the human cases infected with animal assemblages, such as assemblage C in China and Slovakia (Liu et al., 2014; Štrkolcová et al., 2015), assemblage D in German travelers returning from Southeastern Asia (Broglia et al., 2013), and assemblage E in Egypt, Brazil, and Australia (Abdel-Moein and Saeed, 2016; Fantinatti et al., 2016; Zahedi et al., 2017), and assemblage F in Slovakia (Pipiková et al., 2020).Given the presence of these assemblages in humans, which were previously thought to be specific to animal hosts, it is crucial to uncover these modes of transmission in order to improve control measures for *Giardia* infection. Transmission of these *Giardia* spp. might have occurred between any number of animals anywhere in the environment through the fecal-oral route after direct or indirect contact with the infective-stage cysts of the organism (Feng and Xiao, 2011).

In northern Vietnam where livestock live closely with humans, understanding of the risk of *Giardia* transmission from animals to humans (or vice versa) is important (Carrique-Mas and Bryant, 2013). As a preliminary work before starting the full-scale research, the community based cross-sectional study was conducted to analyze risk factors for infection with *Giardia* spp.. In this study, the stool samples were collected from the randomly selected residents and *Giardia* spp. was detected by Direct Immunofluorescence Assay (DFA) using microscope. Meanwhile, specific data about risk factors for *Giardia* spp. infection, including demographic, socio-cultural, and environmental variables, such as livestock possession, were collected from each resident. Based on these data, there is a possibility that the residents who tested positive for *Giardia* spp. tended to keep livestock, especially pigs, and cattle, in this study setting. Based on this phenomenon, we suspect that zoonotic transmission of *Giardia* spp. might have occurred in the field. To confirm the probability of zoonotic transmission patterns of *Giardia* spp., it is necessary to identify the *Giardia* genotypes or assemblage from human and animal stools in this study setting (Thompson and Ash, 2016). Molecular epidemiology from genetic sequence data is, therefore, a better available method to study transmission dynamics of *Giardia* spp. in and between human and animal populations (Thompson and Ash, 2016). Therefore, the objective of this study was to determine the genetic diversity of *Giardia* spp. in both humans and livestock to assess the existence of a route of infection between livestock and humans. Our goal was to assess the role of livestock in the epidemiology of human infection in northern Vietnam. No previous contributions have yet determined the *Giardia* assemblage and *Giardia* sub-assemblage diversity from both humans and livestock in northern Vietnam.

## 2 Methods

### 2.1 Study area

**Fig. 1:**
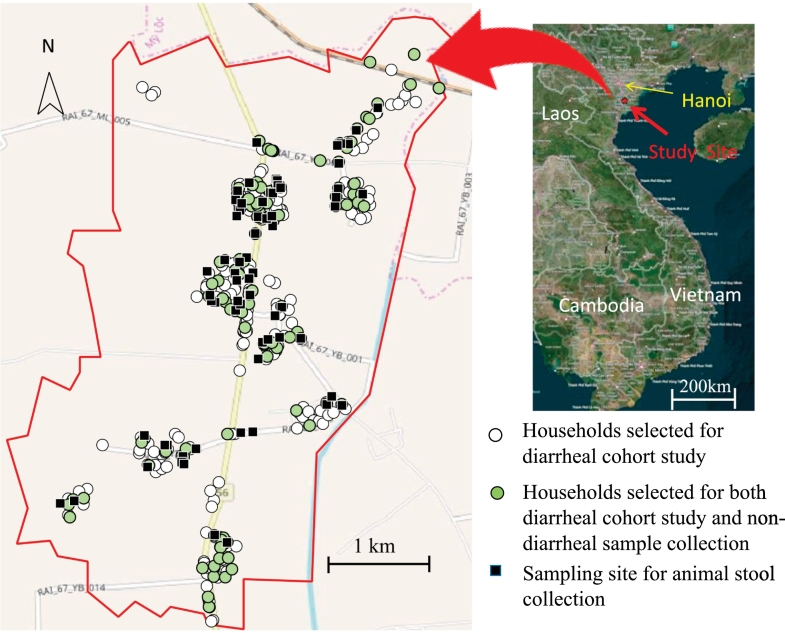
Map of the study area in northern Vietnam. For the diarrheal cohort study, 311 households were randomly selected in this study site (Green circle +White circle). Of these, 105 households were randomly selected for non-diarrheal stool collection (Green circle). Also, animal stool samples were collected (Black square).

This study was carried out in Hien Khanh commune, Vu Ban district, Nam Dinh province, located about 75︎km southeast of Hanoi, Vietnam (Fig. 1). The study area (12 km^2^) included 10 hamlets. The region is part of the Red River Delta of northern Vietnam, with around 10 m elevation. Irrigated rice fields mainly cover this area.

### 2.2 Human diarrheal sample collection (Prospective collection: October 2014–March 2017)

In Hien Khanh commune, there are approximately 2130 households with 8000 residents. Of these households, 435 had children less than 5 years of age accounting for 2225 residents. 311 of the latter households, made up of 1508 residents, were chosen randomly for the diarrheal cohort study, in which diarrheal samples were prospectively collected from all participating residents whenever they suffered from diarrhea, including repeated diarrheal episodes with individual residents (Fig. 1). In this study area, 13 trained health-workers who belonged to the commune health center were organized to visit the enrolled households. Each worker arranged for a twice-weekly visit to approximately 5–20 households to confirm the occurrence of diarrhea and collect diarrheal samples. Sampling was continuously conducted from the end of October 2014 to March 2017. The samples were routinely transported from the study site to the laboratory in Hanoi. The samples, divided for detection of *Giardia* infection, were kept refrigerated at 4 °C prior to examination and were analyzed retrospectively.

### 2.3 Human non-diarrheal sample collection (Cross-sectional collection: September to October 2014)

Of the 311 households selected for “Human diarrheal sample collection”, 105 households were randomly selected for collecting non-diarrheal stool samples (Fig. 1). All 105 households were visited to collect non-diarrheal stool samples in each selected household from all 471 residents. Collection from all selected households was conducted once per household from September to October 2014.

### 2.4 Animal stool sample collection (Cross-sectional collection: August to October 2015)

We revisited the same 105 households selected for “Human non-diarrheal sample collection” from August to October 2015. If the household owned the animal(s), we collected animal stool samples. If we could locate any animals present either in- or outdoors, their fresh stool samples were collected on site (Fig. 1). The specimens included dairy and beef cattle, pigs, and buffalo. Although it is difficult to routinely collect stools from dogs due to their freedom of movement, only dogs kept in sheds or that did not roam freely were targeted for stool samples. The age category of each animal was recorded as adult or infant, when possible. To avoid environmental contamination, the portion of the stool that had not touched to the ground was collected. Exceptionally, pig stool samples were taken directly from the concrete ground of their sheds owing to the difficulty of identifying respective stools given their close quarters in small sheds. In the sheds, we carefully observed whether 1 or more species of animal was housed inside. When it was difficult to identify individual stool samples, samples were collected from various specimens sharing the same defecation site within a 10-m radius, approximately, so long as there were no other species present. Geographical position of sampling points was recorded using a handheld global positioning system (GPS; Garmin, GPSMAP64s). All samples were kept cool during shipment.

### 2.5 Direct Immunofluorescence Assay (DFA)

Stool samples were sedimented using the formalin-ether sedimentation method known as the Medical General Laboratory (MGL) technique (Uga et al., 2010). DFA was used to detect cysts of *Giardia* spp. under the observation of fluorescent microscope (Uga et al., 2010). Antibodies tagged with fluorescent markers, DyLight488 (ARK Fluor Ab C/G - DyLight488, ARK Resource Co., Ltd.), were added to the stool, and incubated. Visualization under a fluorescent microscope (Eclipse 90i, Nikon Instruments Inc.) showed the *Giardia* spp. cysts as green, glowing, ovoid objects (Centers for Disease Control and Prevention. DPDx, 2020).

### 2.6 DNA extraction and molecular analysis

DNA was extracted from sediment samples using a PowerSoil DNA Isolation Kit (MoBio Laboratories Inc., Carlsbad, California) following the manufacturer's instructions, incorporating an initial step of 10 freeze-thaw cycles (freezing in liquid nitrogen for 5 min and heating at 95 °C for 5 min). Elution was accomplished by adding a reduced volume of solution C6 (10 mM Tris) to obtain a final volume of 50 ul and the DNA was stored in a −20 °C freezer. To avoid cross contamination between animal and human samples, each sample was separately detected. PCR amplification and sequence of *Giardia* spp. at the *beta-giardin* (*bg*)*, glutamate dehydrogenase* (*gdh*), and *triose phosphate isomerase* (*tpi*) gene was carried out. Details of primers and cycling conditions are listed in Table 1.

**Table 1:** Primers used to detect and to sequence *Giardia* spp.

Target gene		Primer name	Nucleotide sequence (5′ to 3′)	Polarity	Product (bp)	Cycle condition	Reference
*gdh*	1st PCR	GDHeF	TCA ACG TYA AYC GYG GYT TCC GT	forward	393 bp	95 °C, 5 min, 1 cycle; 94 °C, 30 s, 50 °C, 30 s, 72 °C, 1 min, 45 cycles; 72 °C, 7 min, 1 cycle	Read et al. (2004)
		GDHiR	GTT RTC CTT GCA CAT CTC C	reverse			
	2nd PCR	GDHiF	CAG TAC AAC TCY GCT CTC GG	forward		95 °C, 5 min, 1 cycle; 94 °C, 30 s, 60 °C, 30 s, 72 °C, 1 min, 45 cycles; 72 °C, 7 min, 1 cycle	
		GDHiR	GTT RTC CTT GCA CAT CTC C	reverse			
*tpi*	1st PCR	AL3543	AAA TIA TGC CTG CTC GTC G	forward	490 bp	94 °C, 5 min, 1 cycle; 94 °C, 45 s, 50 °C, 45 s, 72 °C, 1 min, 45 cycles; 72 °C, 10 min, 1 cycle	Sulaiman et al. (2003)
		AL3546	CAA ACC TTI TCC GCA AAC C	reverse			
	2nd PCR	AL3544	CCC TTC ATC GGI GGT AAC TT	forward			
		AL3545	GTG GCC ACC ACI CCC GTG CC	reverse			
*bg*	1st PCR	G7	AAG CCC GAC GAC CTC ACC CGC AGT GC	forward	475 bp	94 °C, 5 min, 1 cycle; 94 °C, 30 s, 65 °C, 30 s, 72 °C, 60 s, 45 cycles; 72 °C, 7 min, 1 cycle	Caccio et al. (2002)
		G759	GAG GCC GCC CTG GAT CTT CGA GAC GAC	reverse			
	2nd PCR	G99	GAA CGA ACG AGA TCG AGG TCC G	forward		94 °C, 5 min, 1 cycle; 94 °C, 30 s, 55 °C, 30 s, 72 °C, 60 s, 45 cycles; 72 °C, 7 min, 1 cycle	
		G609	CTC GAC GAG CT TCG TGT T	reverse			

Note: Each 25 uL reaction mixture contained: GoTaq Green Master Mix (containing Go Taq® DNA Polymerase, dNTP mixture, Green Go Taq Reaction Buffer, MgCl2; Promega) with 5% dimethyl sulfoxide (Sigma–Aldrich, USA) and 0.4 mg/ml BSA (Sigma-Aldrich, USA).

PCR assays were performed on the MyCycler thermal cycler (Bio-Rad, Hercules, USA) using the GoTaq Green Master Mix (Promega). In all the PCR reactions, a Giardia-positive DNA specimen, and distilled water were used as positive and negative controls. Amplified DNA products were evaluated by 1.5% agarose gel electrophoresis. For all positive samples, products of the expected size were gel-extracted and purified using a MonoFas DNA Purification Kit (GL Sciences, Tokyo, Japan), and were sequenced in both directions with an ABI 3130 genetic analyzer (Applied Biosystems, Foster City, CA, USA) using the secondary PCR primers and a BigDye Terminator V3.1 cycle sequencing kit (Applied Biosystems, Foster City, CA, USA). Nucleic acid sequences were compared with those in the GenBank database using the BLAST program. Sequences from this study were deposited in the GenBank. Phylogenetic analysis was performed in MEGA 6 using neighbor-joining (NJ) algorithms with evolutionary distances calculated using the Kimura-2 parameter method (Kimura, 1980) with bootstrap value of 1000. Sequences including heterozygous (di-nucleotide) sites were excluded from the analyses in order to avoid distorting the topology of the phylogenetic trees. The nucleotide sequences retrieved from the GenBank to construct the phylogenetic tree are listed in Supplementary Table S1.

### 2.7 Definition of positive samples

Human stool samples positive for *Giardia* spp. were defined using a 2-step method; (1) all samples were screened with DFA, (2) only positive samples with DFA were screened with PCR and defined positive using either the *bg* or *gdh* gene, or both. Human samples detected negative by DFA were not available to extract DNA due to the considerable effort dedicated to analyzing the assemblages of samples already detected positive for estimation of transmission.

Unlike with human stool samples, DNA extraction was carried out for all animal samples due to the considerable effort dedicated to PCR analysis, in order to reduce the time-consuming microscopic detection of animal stools with their many impurities. Animal stool samples positive for *Giardia* spp. were defined through PCR analysis using either the *bg* or *gdh* gene, or both.

### 2.8 Ethics statement

This study was approved by the Ethical Committee of the Graduate School of International Health and Development, Nagasaki University and approved by the Institutional Review Board of NIHE in Vietnam (Approved No.130925115-2). Written informed consent was obtained from participants who were the head of household for each household. Verbal consent by livestock owners was obtained prior to the collection of fecal samples from private land.

The participants were notified and understood that the test results of their samples would not be available to assist in treatment. However, they would be monitored for *Giardia* clinical manifestations and other diarrheal symptoms by trained health-workers, and were advised to report any abnormal health status to the local physician in the Hien Khanh Commune Health Station, which was staffed with three physicians, one pharmacist, one midwife, and one nurse. If any *Giardia* manifestations occurred, the participant would be referred to District health center for further case management. If diarrheal symptoms were present, all participants with diarrhea would be treated according to the Ministry of Health guidelines at the Community health center. Severe cases of diarrhea were referred to the District or the Provincial hospital for adequate laboratory testing and treatment.

## 3 Results

### 3.1 Human diarrheal stool samples

**Fig. 2:**
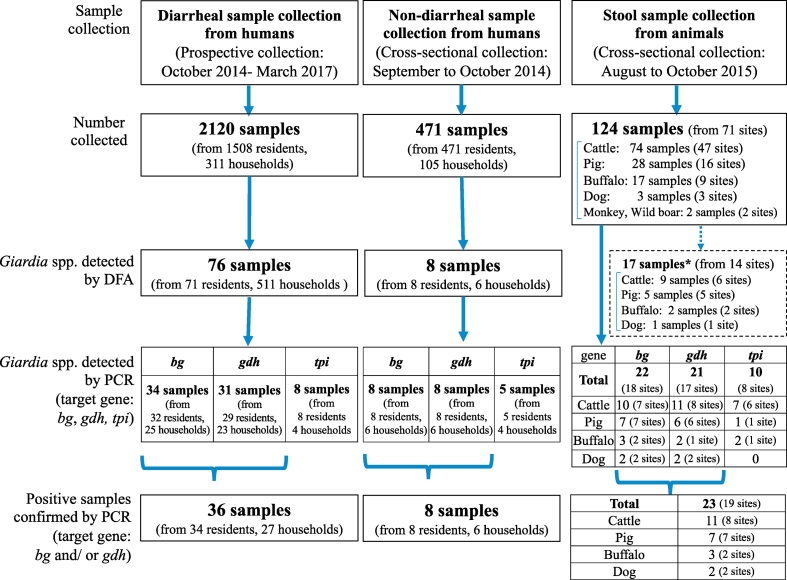
Results of different methods. * We did not use these results because of difficulty in detection of animal samples containing many impurities.

**Fig. 3:**
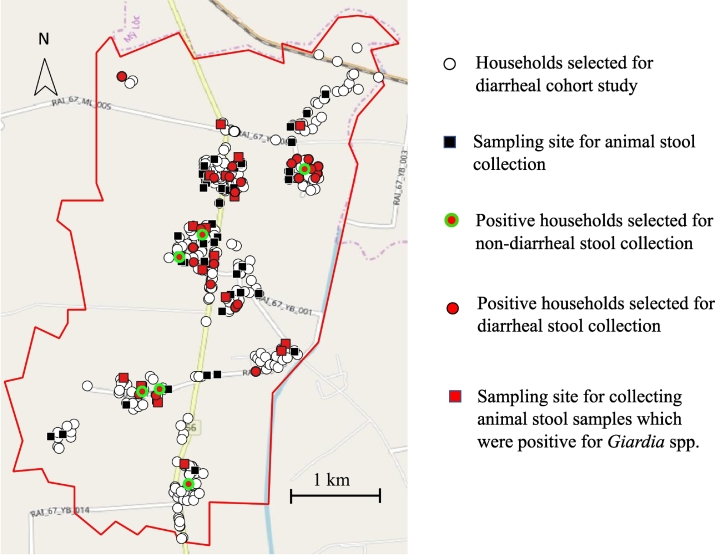
Distribution of sampling sites and households and *Giardia*-positive sites.

A total of 2120 diarrheal samples from a total 1508 residents in Hien Khanh commune were collected from 2014 to 2017. Using DFA, we observed *Giardia* cyst from 76 samples by a fluorescent microscope. Of these, 36 samples were confirmed positive through the definition by PCR using either the *bg* or *gdh* gene, or both (Table 2, Fig. 2). Only 8 samples amplified successfully with *tpi* gene (Table 2). All samples detected by PCR using the *tpi* gene were also detected by PCR using the *bg* and *gdh* genes. Distribution of positive samples is depicted in Fig. 3.

**Table 2:** Genotype characterization of *Giardia* spp. isolates from human diarrheal samples.

Sample ID	Number of mixed individuals	Genotype (GenBank Accession No.)		
		*bg*	*gdh*	*tpi*
CDS_0256	1	E (AY653159.1), E (LC503938)⁎ B (DP)	B (KC313934.1), B (DP)	B (DP)
CDS_0257	1	B (DP)	B (KT124839.1), B (LC430569.1), B (DP)	B (DP)
CDS_0258	1	B (DP)	B (LC430569.1)	B (DP)
CDS_0259	1	E (AY653159.1)	B (LC503954), B(*DP*)x3	B (DP)
CDS_0261	1	B (DP)	B (LC503955), B(DP)	B (DP)x2
CDS_0658	1	E (AY653159.1)	B (LC503956), B (MK043575.1)	.
CDS_0659	1	AII (AY072724.1), E (AY653159.1)	B (KT124839.1), E (AB182127.1)	.
CDS_0660	1	E (LC503939)⁎	B (LC503966)⁎, E (LC503966)⁎	.
CDS_0763	1	AII (LC503945), E (AY653159.1)	.	.
CDS_0992	1	B (LC503950)	B (DP)	.
CDS_1192	1	B (LC503951)⁎	B (AB295651.1)	B (DP)
CDS_1194	1	AII (DP)	.	.
CDS_1205	1	AII (AY072724.1)	B (AB569386.1)	.
CDS_1234	1	B (LC503947)⁎, B (LC503948)⁎	AII (AB195223.1), B (LC503957)⁎	.
CDS_1236	1	B (LC503947)⁎	B (LC503958)⁎	.
CDS_1250	1	AII (LC503942)⁎, B(DP)	AII (LC503952)⁎	.
CDS_1272	1	AII (LC503940)⁎	AII (LC503953)⁎, B (LC503959)⁎	.
CDS_1274	1	B (LC503947)⁎	B (AB569386.1)	.
CDS_1285	1	AII (DP), AII (LC503941)⁎	B (AB569386.1)	.
CDS_1297	1	B (LC503947)⁎, B (DP)	B (DP)	B (LC503967)⁎, B (LC503968)⁎, B (DP)
CDS_1298	1	B (AY072727.1), B (DP)	B (DP)	B (LC503968)⁎, B (DP)
CDS_1308	1	B (LC503947)⁎	B (LC503960)⁎	.
CDS_1368	1	B (AY072727.1)	AII (AB808753.1), B (AB569386.1)	.
CDS_1386	1	AII (DP), B (AY072727.1)	B (AB618784.1)	.
CDS_1392	1	AII (AY072724.1), B (AY072727.1)	B (AB295651.1), B (AB569386.1)	.
CDS_1447	1	AII (AY072724.1), B (DP)	B (LC503961)⁎, B (DP)	.
CDS_1469	1	AII (AY072724.1), B (AY072727.1)	B (LC503962)⁎	.
CDS_1484	1	AII (AY072724.1), AII (LC503944)⁎	B (AY178749.1), B (DP)	.
CDS_1534	1	AII (AY072724.1), B(DP)	B (AB569386.1), B (LC503963)⁎	.
CDS_1660	1	.	B (AB569386.1)	.
CDS_1679	1	AII (LC503943), AII (LC503946)⁎	.	.
CDS_1696	1	B (AY072727.1)	B (DP)	.
CDS_1697	1	B (DP)	B (LC503964)⁎	.
CDS_1698	1	.	B (LC503965)⁎	.
CDS_1739	1	AII (AY072724.1)	.	.
CDS_1921	1	B (LC503949)⁎	.	.

DP: GenBank accession number was not shown due to sequences with double peak.

⁎ The novel sequences without heterogeneous positions were newly submitted to GenBank.

### 3.2 Human non-diarrheal stool samples

A total of 471 non-diarrheal stool samples from residents in Hien Khanh commune were collected in 2014. A total of 8 samples were detected as positive for *Giardia* spp. by DFA. Also, they were successful in DNA extraction and were found positive through PCR using *bg* and *gdh* genes (Table 3, Fig. 2). Only 5 samples amplified successfully with the *tpi* gene (Table 3). All samples detected by PCR using the *tpi* gene were also detected by PCR using the *bg* and *gdh* genes. Distribution of positive samples is depicted in Fig. 3.

**Table 3:** Genotype characterization of *Giardia* spp. isolates from human non-diarrheal samples.

Sample ID	Genotype (GenBank Accession No.)		
	*bg*	*gdh*	*tpi*
63_5	BIII (AY072727.1),	B (AB295651),	B (AY228628.1),
	E (AY653159.1)	B (DP),	B(LC503770)⁎
		E (AB182127.1)	
63_6	E (AY653159.1)	B (LC504285)⁎,	.
		E (AB182127.1)	
63_7	BIII (AY072727.1)	B (AB295651),	B (AY228628.1),
		B (LC504286)⁎,	B (LC503771)⁎
		B (DP)	
179_2	AII (AY072724.1)	A (AB195223.1)	AII (AY368157.1)
800_2	E (AY653159.1)	B (AB295651),	.
		B (AY178749.1)	
1160_6	E (AY653159.1)	B (AB295651),	B (KF922912.1)
		E (AB182127.1)	
1172_4	AII (AY072724.1)	A (AB195223.1)	AII (AY368157.1)
1245_7	E (AY653159.1)	B (AB295651),	.
		E (AB182127.1)	

DP: GenBank accession number was not shown due to sequences with double peak.

⁎ The novel sequences without heterogeneous positions were newly submitted to GenBank.

### 3.3 *Giardia* spp. in livestock

From August 2015 to October 2015, a total of 124 samples from 71 sampling sites were collected from livestock (Fig. 2). All animals were healthy at the time of sampling. All samples were examined by DFA and PCR targeting *bg, gdh*, and *tpi* genes. Of these samples, 22 were positive using the *bg* gene, 21 samples were positive using the *gdh* gene, and 10 samples were positive using the *tpi* gene. Although PCR analysis of the *bg* and *gdh* genes had a higher detection rate, 17.4% (22/124) and 17.7% (21/124), respectively, those of the *tpi* gene provided a lower rate of detection; 8.1% (10/124) (Table 4, Fig. 2). All samples detected by PCR using the *tpi* gene were also detected by PCR using the *bg* and *gdh* genes.

**Table 4:** Genotype characterization of *Giardia* spp. isolates from animal stool samples.

Sample ID	Animal	Number of mixed individuals	Genotype (GenBank Accession No.)		
			*bg*	*gdh*	*tpi*
Ani_15	Buffalo	1	E (AY653159.1)	.	.
Ani_55	Buffalo	1	AIII (LC503598)⁎	AIII (DQ100288.1)	AIII (LC503768)⁎
Ani_56	Buffalo	1	AIII (LC503598)⁎	AIII (DQ100288.1)	AIII (LC503768)⁎
Ani_11	Cattle	1	E (AY653159.1)	E (AB182127.1)	E (LC503769)⁎
Ani_32	Cattle	2	E (AY653159.1)	E (AB182127.1)	.
Ani_33	Cattle	1	E (AY653159.1)	E (AB182127.1)	.
Ani_40	Cattle	1	E (AY653159.1)	E (AB182127.1)	E (AY655705)
Ani_48	Cattle	1	AII (AY072724.1)	E (LC504280)⁎	.
Ani_58	Cattle	1	E (AY653159.1)	E (AB182127.1)	EII (AY655705)
Ani_80	Cattle	1	.	E (LC504281)⁎	.
Ani_90	Cattle	1	E (AY653159.1)	E (AB182127.1)	EII (AY655705)
Ani_114	Cattle	1	E (AY653159.1)	E (AB182127.1)	EII (AY655705)
Ani_146	Cattle	1	E (AY653159.1)	E (AB182127.1)	EII (AY655705)
Ani_147	Cattle	1	E (AY653159.1)	E (AB182127.1)	EII (AY655705)
Ani_7	Dog	3	E (AY653159.1)	E (AB182127.1)	.
Ani_140	Dog		E (AY653159.1)	E (AB182127.1)	.
Ani_6	Pig	1	E (AY653159.1)	E (LC504283)⁎	.
Ani_14	Pig	12	E (AY653159.1)	E (AB182127.1)	.
Ani_19	Pig	1	E (AY653159.1)	E (LC504284)⁎	.
Ani_23	Pig	1	AII (AY072724.1), AII (LC503600)⁎, E (AY653159.1), E (DP), E (LC503601) ⁎	E (AB182127.1)	.
Ani_42	Pig	1	AII (LC503599)⁎	.	.
Ani_47	Pig	1	A (DP), E3 (AY653159.1)	E (LC504282)⁎	.
Ani_73	Pig	10	E (AY072729.1)	E (AY178741.1)	E (KJ668136.1)

DP: GenBank accession number was not shown due to sequences with double peak.

⁎ The novel sequences without heterogeneous positions were newly submitted to GenBank.

**Buffalo**: We confirmed 3 samples from 2 sampling sites were positive for *Giardia* spp. (Supplementary Fig. S1).

**Cattle**: We confirmed 11 samples from 8 sampling sites were positive for *Giardia* spp. (Supplementary Fig. S2).

**Pig**: We confirmed 7 samples from 6 sampling sites were positive for *Giardia* spp. (Supplementary Fig. S3).

**Dog**: We confirmed 2 samples from 2 sampling sites were positive for *Giardia* spp. (Supplementary Fig. S4).

**Other animals**: We collected stool samples from 1 monkey and 1 boar, which were kept in their sheds. There was no *Giardia* spp. detected.

### 3.4 Genotyping analysis of *Giardia* spp. isolates at the *bg* gene

High-quality sequencing data of the *bg* gene were available for the 85 isolates. According to the alignment analysis of the *bg* gene, 26 isolates (2 buffalo, 3 pig, 1 cattle, 2 human non-diarrheal, 18 human diarrheal samples) were characterized as assemblage A; 27 isolates (2 human non-diarrheal, 25 human diarrheal samples) were characterized as assemblage B; 32 isolates (5 human non-diarrheal, 7 human diarrheal, 1 buffalo, 9 cattle, 8 pig, 2 dog samples) were characterized as assemblage E. Through multiple re-amplification and re-sequencing efforts, 18 samples were found to have mixed infection (Ani_23, Ani_47, 63_5, CDS_256, CDS_659, CDS_763, CDS_1234, CDS_1250, CDS_1285, CDS_1297, CDS_1298, CDS_1386, CDS_1392, CDS_1447, CDS_1469, CDS_1484, CDS_1534, CDS_1679). These mixed infections not only derived from different assemblages, but also within assemblages. Of these, 12 mixed infections (Ani_23, Ani_47, 63_5, CDS_256, CDS_659, CDS_763, CDS_1250, CDS_1386, CDS_1392, CDS_1447, CDS_1469, CDS_1534) were from different assemblages, 8 mixed infections (Ani_23, CDS_256, CDS_1234, CDS_1285, CDS_1297, CDS_1298, CDS_1484, CDS_1679) were found within assemblage.

**Fig. 4:**
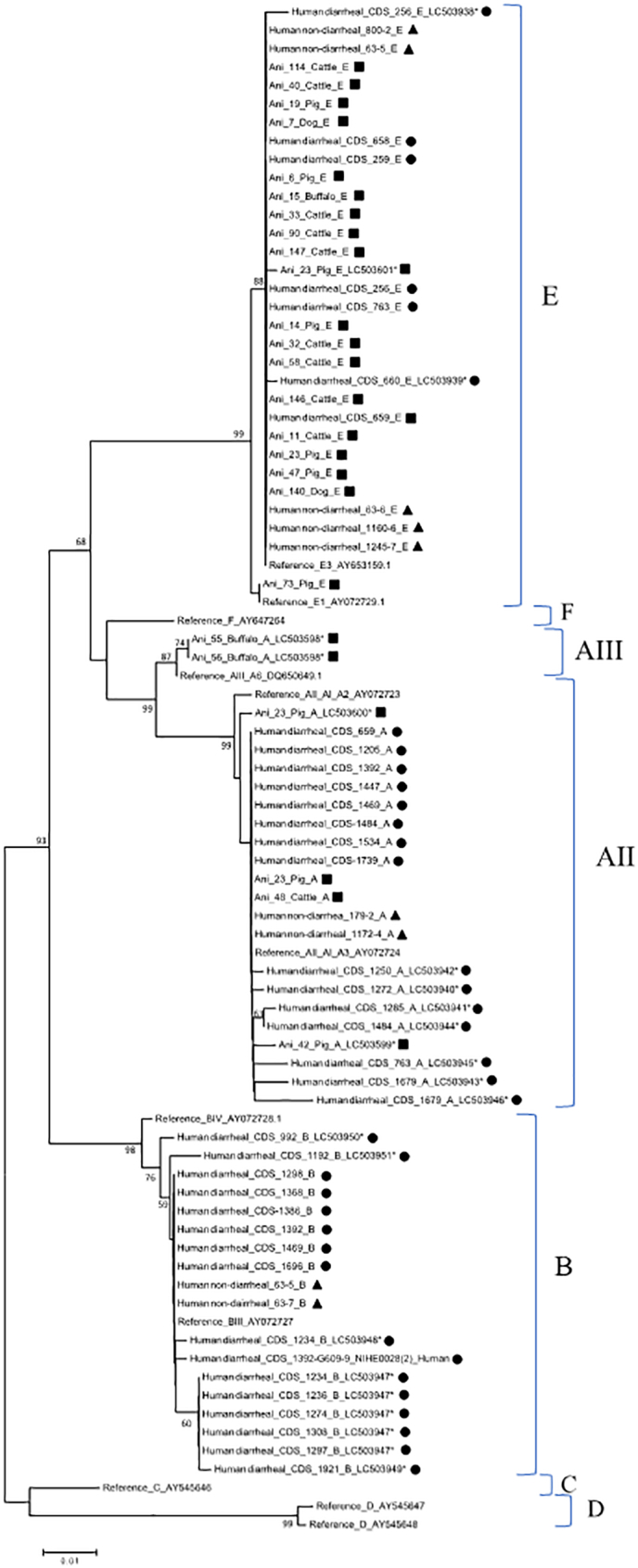
Phylogenetic analysis of partial *bg* sequences. Filled squares, circle, and triangles represent animal, human diarrheal, and human non-diarrheal samples, respectively, from this study. Bootstrap values were obtained using 1000 pseudo-replicates and those greater than >50% are shown on nodes. The evolutionary distances were computed using the Kimura-2 parameter method. GenBank accession numbers are provided for representative sequences. Symbol (*) indicates novel sequences newly submitted to GenBank.

Phylogenetic analysis revealed the presence of 2 sub-assemblages of assemblage A (Fig. 4). One sub-assemblage (24 isolates) was characterized as AII and the other sub-assemblage (2 isolates) was characterized as AIII. Out of 24 isolates of *Giardia* spp. assigned to the sub-assemblage AII, 12 isolates (1 pig, 1 cattle, 2 human non-diarrheal, and 8 diarrheal samples) exhibited 100% identity with the reference sequence AY072724.1 between position 111 and 585. The remaining 12 isolates differed by 1 to 5 SNPs from the sequence AY072724.1 (Supplementary Table S2). Similarly, 2 isolates (2 buffalo) assigned to the sub-assemblage AIII differed by 1 SNP from the sequence DC650649.1 (Supplementary Table S2).

In terms of assemblage B, phylogenetic analysis revealed the presence of a cluster containing 2 sub-assemblages, BIII and BIV (Fig. 4). Out of 27 isolates, 8 isolates (2 human non-diarrheal, 6 human diarrheal) exhibited 100% identity with the reference sequence AY072727.1 between position 111 and 585, while the remaining 19 isolates differed by 1 to 10 SNPs from the sequence AY072727.1 (Supplementary Table S3).

Furthermore, the presence of 2 sub-types of *Giardia* spp. assemblage E was also revealed by phylogenetic analysis (Fig. 4). Out of 32 isolates, 1 sub-type (31 isolates) was characterized as E3 and the other sub-type (1 isolate) was characterized as E1. Out of 31 isolates of *Giardia* spp. assigned to the sub-type E3, 27 isolates exhibited 100% identity with the reference sequence for assemblage AY653159.1 (E3) between position 18 and 492, and 1 isolate (Ani_73) exhibited 100% identity with the reference sequence AY072729.1 (E1). The remaining 4 isolates (Ani_23 x2, CDS_256, CDS_660) differed by 1 to 4 SNPs from the reference sequence of AY653159.1 (Supplementary Table S4).

### 3.5 Genotyping analysis of *Giardia* spp. isolates at the *gdh* gene

High quality sequencing data of the *gdh* gene were available for the 84 isolates. According to the alignment analysis of the *gdh* gene, 8 isolates (2 buffalo, 2 human non-diarrheal, 4 human diarrheal) were characterized as *Giardia* spp. assemblage A; 51 isolates (10 human non-diarrheal, 41 human diarrheal) were characterized as *Giardia* spp. assemblage B; 25 isolates (4 human non-diarrheal, 2 human diarrheal, 11 cattle, 6 pig, 2 dog) were characterized as *Giardia* spp. assemblage E. Through multiple re-amplification and re-sequencing efforts, 20 samples were found to have mixed infection (63_5, 63_6, 63_7, 800_2, 1160_2, 1245_7, CDS_256, CDS_257, CDS_259, CDS_261, CDS_658, CDS_659, CDS_660, CDS_1234, CDS_1272, CDS_1368, CDS_1392, CDS_1447, CDS_1484, CDS_1534). Of these, 9 mixed infection (63_5, 63_6, 1160_6, 1245_7, CDS_659, CDS_660, CDS_1234, CDS_1272, CDS_1368,) were different assemblage, 12 mixed infection (63_5, 63_7, 800_2, CDS_256, CDS_257, CDS_259, CDS_261, CDS_658, CDS_1392, CDS_1447, CDS_1484, CDS_1534) were within assemblage.

**Fig. 5:**
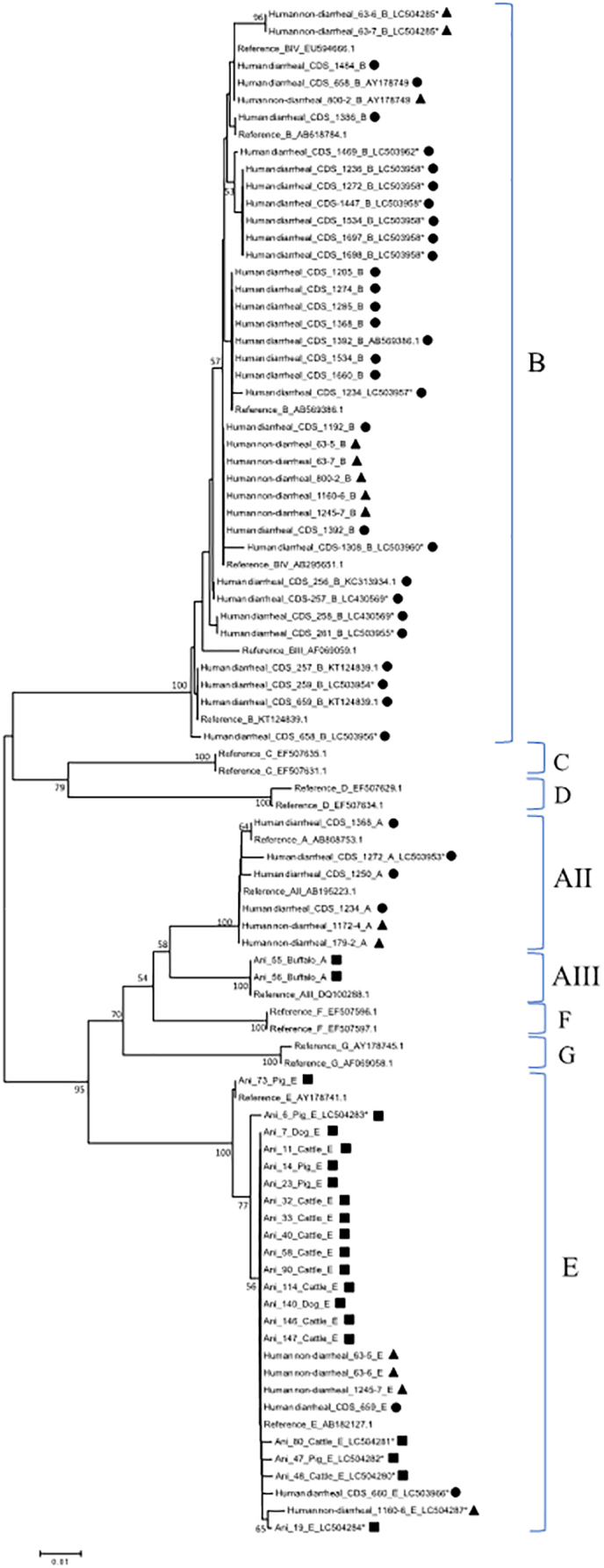
Phylogenetic analysis of partial *gdh* sequences. Filled squares, circle, and triangles represent animal, human diarrheal, and human non-diarrheal samples, respectively, from this study. Bootstrap values were obtained using 1000 pseudo-replicates and those greater than >50% are shown on nodes. The evolutionary distances were computed using the Kimura-2 parameter method. GenBank accession numbers are provided for representative sequences. Symbol (*) indicates novel sequences newly submitted to GenBank.

Phylogenetic analysis revealed the presence of 2 sub-assemblages of *Giardia* spp. assemblage A (Fig. 5). One sub-assemblage (6 isolates) was characterized as AII and the other sub-assemblage (2 isolates) was characterized as AIII. Out of 6 isolates of *Giardia* spp. assigned to the sub-assemblage AII, 3 isolates (179-2, 1172-4, CDS_1234) exhibited 100% identity with the reference sequence AB195223.1 between position 48 and 440. The remaining 3 isolates differed by 1 to 2 SNPs from the sequence AB195223.1. Similarly, 2 isolates assigned to the sub-assemblage AIII exhibited 100% identity with the reference sequence DQ100288.1 (Supplementary Table S5).

In terms of assemblage B, phylogenetic analysis revealed the presence of a cluster containing 2 sub-assemblages, BIII and BIV (Fig. 5). Out of 51 isolates, 6 isolates (4 human non-diarrheal, 2 human diarrheal) exhibited 100% identity with the reference sequence AB295651.1 between position 1 and 393. The remaining 45 isolates differed by 1 to 5 SNPs from the sequence AB295651.1 (Supplementary Table S6).

Furthermore, the presence of assemblage E was also revealed by phylogenetic analysis (Fig. 5). Out of 25 isolates, 17 isolates (9 Cattle, 2 Dog, 2 Pig, 3 human non-diarrheal, 1 human diarrheal) exhibited 100% identity with the reference sequence for assemblage AB182127.1 between position 48 and 440, and 1 isolate (Ani_73) exhibited 100% identity with the reference sequence AY178741.1. The remaining 7 isolates (Ani_6, Ani_80, Ani_47, Ani_48, 1160–6, Ani_19, CDS_660) differed by 1 to 2 SNPs from the reference sequence of AY182127.1(Supplementary Table S7).

### 3.6 Genotyping analysis of *Giardia* spp. isolates at the *tpi* gene

High quality sequencing data of the *tpi* gene were available for the 28 isolates. According to the alignment analysis of the *tpi* gene, 8 isolates (2 buffalo, 2 human non-diarrheal) were characterized as assemblage A; 16 isolates (5 human non-diarrheal, 11 human diarrheal) were characterized as assemblage B; 8 isolates (7 cattle, 1 pig) were characterized as assemblage E. Through multiple re-amplification and re-sequencing efforts, 4 samples were found with mixed infection within assemblage B (63_5, 63_7, CDS_1297, CDS_1298).

**Fig. 6:**
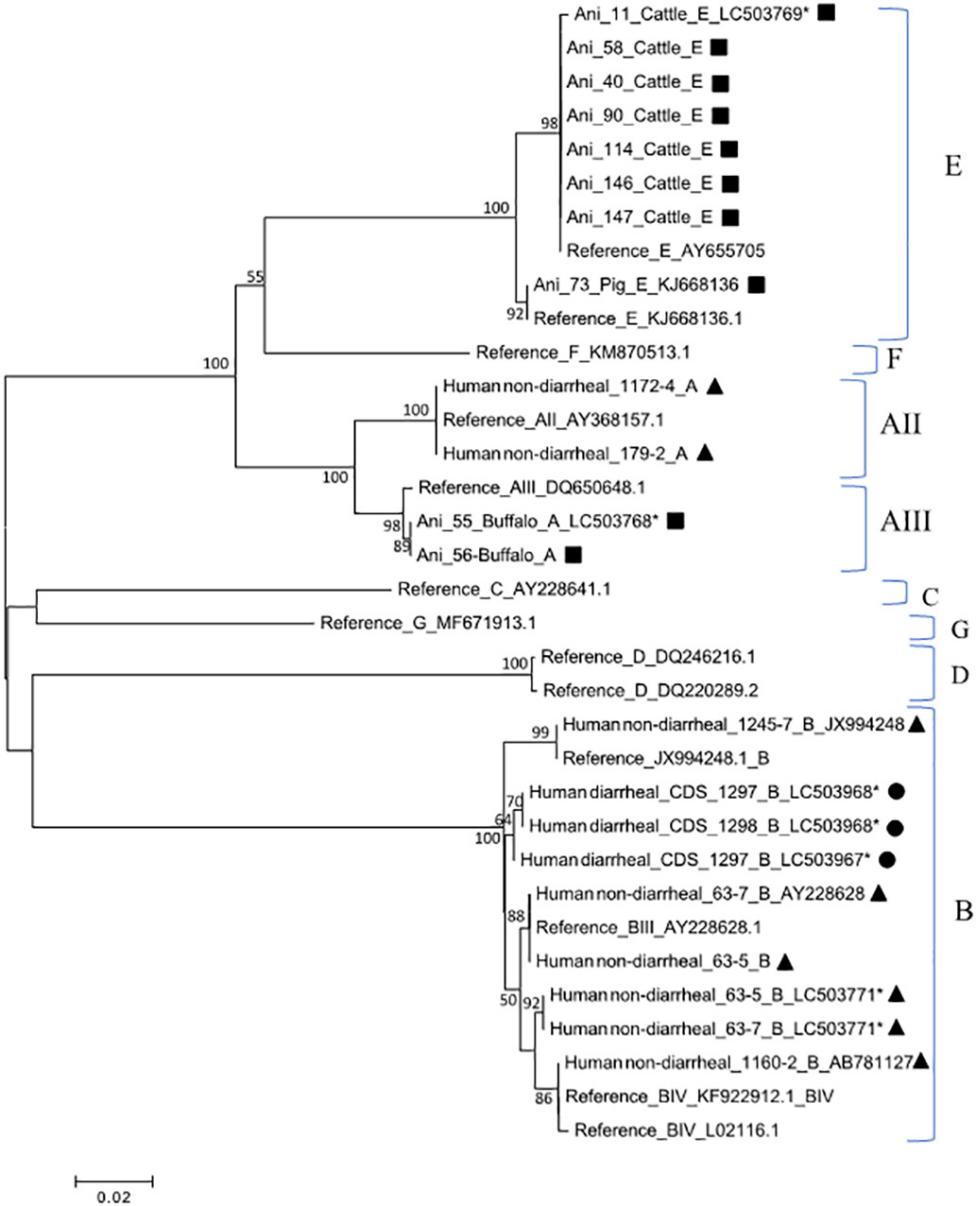
Phylogenetic analysis of partial *tpi* sequences. Filled squares, circle, and triangles represent animal, human diarrheal, and human non-diarrheal samples, respectively, from this study. Bootstrap values were obtained using 1000 pseudo-replicates and those greater than >50% are shown on nodes. The evolutionary distances were computed using the Kimura-2 parameter method. GenBank accession numbers are provided for representative sequences. Symbol (*) indicates novel sequences newly submitted to GenBank.

Phylogenetic analysis revealed the presence of 2 sub-assemblages of assemblage A (Fig. 6). One sub-assemblage (2 isolates from human non-diarrheal samples) was characterized as AII, which exhibited 100% identity with the reference sequence AY368157.1 between position 22 and 511. The other sub-assemblage (2 isolates from 2 buffalo samples) was characterized as AIII, which differed by 1 SNP from the reference sequence of DQ650648.1 (Supplementary Table S8).

In terms of assemblage B, phylogenetic analysis revealed the presence of a cluster containing 2 sub-assemblages of *Giardia* spp. sub-assemblage BIII and BIV (Fig. 6). Out of 19 isolates, 2 isolates (2 human non-diarrheal) exhibited 100% identity with the reference sequence AB228628.1 between position 22 and 511. The remaining 17 isolates differed by 4 to 12 SNPs from the sequence AB228628.1 (Supplementary Table S9).

Furthermore, the presence of assemblage E was also revealed by phylogenetic analysis (Fig. 6). Out of 8 isolates, 6 isolates (6 Cattle) exhibited 100% identity with the reference sequence for assemblage AY655705.1 between position 9 and 498; 1 isolate (Ani_73) exhibited 100% identity with the reference sequence KJ668136.1; and the remaining isolate (Ani_11) differed by 1 SNP from the reference sequence of AY655705.1 (Supplementary Table S10).

The novel sequences without heterogeneous positions detected in the present study are available in the GenBank database under accession numbers LC503598-LC503601, LC503768-LC503771, LC503938-LC503973, and LC504280-LC504287.

### 3.7 Zoonotic transmission potential of *Giardia* spp.

**Fig. 7:**
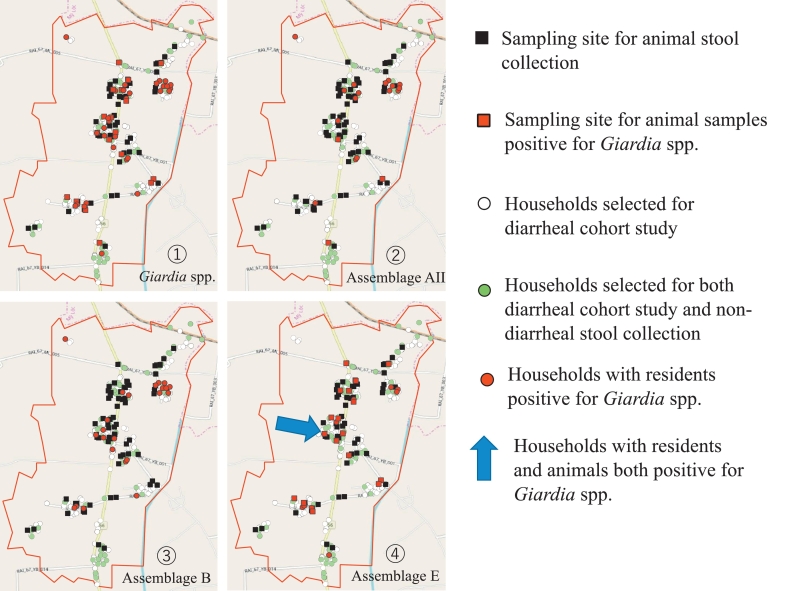
Distribution of *Giardia*-positive samples among all samples, including animal and human stool samples. ① The red shows positive sites for all assemblages ② only assemblage AII ③ only assemblage B ④ only assemblage E; The blue arrow shows the specific multiple cases in one household. (For interpretation of the references to colour in this figure legend, the reader is referred to the web version of this article.)

Our data indicated that assemblage AII and E, which were detected in both animal and human samples, were widely distributed throughout our study site (Fig. 7). The arrow in Fig. 7④ shows the specific multiple cases of *Giardia*-positive occurrences in 1 household. In this house, 7 human non-diarrheal samples, 4 cattle samples, and 1 pig sample (Ani_32, Ani_33, Ani_35, Ani_34) were collected. Of these, 7 samples (3 human non-diarrheal samples; 63–5, 63–6, 63_7, and 4 cattle samples; Ani_32, Ani_33, Ani_146, Ani_147) were positive for *Giardia* spp. In the *bg* gene, 6 samples (3 human non-diarrheal sample; 63_5, 63_6, 63_7, and 3 cattle samples; Ani_32, Ani_146, Ani_147) were positive in the *gdh* gene. Of the 7 positive samples in the *bg* gene, identical sequence results of assemblage E were obtained from 2 human non-diarrheal samples (63_5, 63_6) and 4 cattle samples (Ani_32, Ani_33, Ani_146, Ani_147). Of the 6 positive samples in the *gdh* gene, identical sequence results of assemblage E were obtained from 2 human non-diarrheal samples (63_5, 63_6) and 3 cattle samples (Ani_32, Ani_146, Ani_147). Assemblage B was also isolated from human samples, but there were no positive animal samples. According to the definition of *Giardia*-positive, we identified the positive samples from humans and animals that had the exact same sequences of assemblage E at both the *bg* and *gdh* genes except at the *tpi* gene. This suggests evidence of cross-species transmission between cattle and humans.

## 4 Discussion

Although the potentiality of zoonotic transmission of *Giardia* infections remains controversial, this study provides information on the genetic diversity of *Giardia* spp. among humans and livestock in an agricultural area of Vietnam. According to our results, assemblage A and E have been detected in a wide range of animals, such as buffalo, cattle, pigs, as well as in humans, while assemblage B appeared only in humans. In this study, assemblage A was categorized in 2 sub-assemblages; one is sub-assemblage AII, which is commonly found in humans (Avendaño et al., 2019; Faria et al., 2017; Hernández et al., 2019; Lecová et al., 2018; Naguib et al., 2018), although it has also been reported in a number of studies in animals; and the other sub-assemblage AIII is mainly found in animals (Feng and Xiao, 2011; Ryan and Cacciò, 2013; Thompson and Ash, 2016). Our phylogenetic analysis could also clearly distinguish assemblage AIII from assemblage AII. This analysis reveals that sub-assemblage AIII was common only in buffalo among the targeted animals for this study in Nam Dinh province. However, our findings suggest that sub-assemblage AII may be widespread pathogens transmitted between or among humans and animals, such as cattle, dogs, pigs, and buffalo. Strikingly, in this study, assemblage E was detected not only in animals, but also in humans. This suggests the first human infection case of assemblage E in Vietnam.

Specifically, the multiple cases identified in one particular household (household 0063) confirms zoonotic transmission of *Giardia* spp. assemblage E between bovine and human hosts. However, due to limitations in sampling design, we could not confirm any household in which assemblage A (AII) was simultaneously detected in animals and humans. Nevertheless, our study confirms that assemblage E and AII were detected in both animal and human samples, including in non-diarrheal and diarrheal samples, in the study site. However, assemblage AIII was only detected in buffalo but not in humans and other animals. In this study site, assemblage AII and E are presumably transmitted between livestock and humans through direct physical contact with each other or with manure, and/or through indirect contact with environmental contamination of food, water, or soil.

Although evidence for zoonotic transmission of assemblage B was not detected in this study area, the frequent detection of assemblage B in human diarrheal samples was notable. Not only detecting the zoonotic potential, but also understanding the pathogenesis is vital. Studies have suggested that assemblage B causes diarrhea and has higher pathogenicity than assemblage A (Einarsson et al., 2016; Flecha et al., 2015; Gelanew et al., 2007). While some studies have reported a significant association of assemblage A with diarrhea (Haque et al., 2005; Sahagún et al., 2008), it remains uncertain whether differences of assemblage, are associated with differences in pathogenesis (Thompson and Smith, 2011).

Our study has several limitations to confirm our study objectives. Firstly, it was difficult to perfectly match different sampling methods, such as human diarrheal sampling, human non-diarrheal sampling, and animal stool sampling. Our prospective diarrheal cohort study was performed between September 2014 and March 2017. During this period, human diarrheal samples were continually collected from all participating residents from all 311 households whenever they suffered from diarrhea, including repeated collection of samples. However, non-diarrheal samples were cross-sectionally collected from 105 randomly selected households in September 2014. Also, animal stool samples were cross-sectionally collected in September 2015 after having finished stool sampling from residents. In this way, animal samples and human non-diarrheal samples were not collected during the same period. Moreover, no obvious differences in frequency of *Giardia* spp. were determined between each species of animal and humans as some animal samples originated from multiple individuals, especially samples collected from pigpens. Our study objectives did not include directly comparing the detection rate or detection date of *Giardia* spp. between diarrheal and non-diarrheal samples or human and animal samples, therefore transmission among humans or between humans and animals may have been underestimated. If we had been able to collect fecal samples from animals or/and humans immediately after the detection of *Giardia* spp., we might have been able to identify more cases for zoonotic transmission.

Secondly, the infection routes of *Giardia* spp. (assemblage E) between livestock and humans were solely based upon the results from non-diarrheal samples. We presumed that residents of one household (household_0063) who tested positive for *Giardia* spp. (assemblage B and E) from non-diarrheal samples were likely to suffer from diarrhea during our prospective cohort study. However, there were ultimately no cases of diarrheal episodes in this household, although the occurrence of diarrheal episodes was carefully tracked throughout the study period. Some researchers have suggested that most *Giardia* spp. infections are chronic and asymptomatic (Einarsson et al., 2016; Kotloff et al., 2013). At least in the case of Household 0063, transmission of *Giardia* spp. from animals to humans may not directly cause human diarrhea. However, this data does not rule out transmission between humans and animals.

Thirdly, there is the difficulty of DNA extraction from stool samples and the problem of DNA degradation. In terms of human samples, including both diarrheal and non-diarrheal samples, DNA extraction was conducted from only *Giardia* spp. positive samples detected by DFA using microscopy due to the large sample size. In terms of animal stool samples, we extracted DNA from all samples collected regardless of the results of microscopic detection. Not a few animal stool samples that were DFA positive for *Giardia* spp. were negative through PCR, and vice versa. It is very difficult to compare the accuracy of DFA-based results versus PCR-based results. There are many factors that affect the results of DFA and PCR. The methods of DFA are known to cross-react with non-target organisms such as algae (Rodgers et al., 1995), thereby providing false positive microscopy results, especially for animal stool samples. Alternatively, false negative PCR amplification may arise due to the inhibitory effects of substances such as humic acids, which are present in sewage/environmental samples (Mayer and Palmer, 1996). Also, there are differences in efficiency of PCR amplification between different genes (Ryan and Cacciò, 2013). In this study, the success of PCR amplification using the primer set of the *tpi* gene was lower compared to that of the *bg* and *gdh* genes. Even though DNA was successfully extracted as proved by sequencing the *Giardia bg* genes, the quantity of DNA was low. This was problematic when we repeated PCR analyses using different targeted genes for reliable consistent interpretation. Also, multiple re-amplification and re-sequencing efforts using the same targeted gene revealed numerous mixed infections, especially in human samples. Entire genome analysis using next generation sequencing can provide a deeper understanding in the identification of more variable genes (Thompson and Ash, 2019). Unfortunately, the amount of DNA extracted in this study was not sufficient for this analysis. As a consequence, genetic diversity may have been underestimated in this study.

Molecular characterization of *Giardia* spp. in fecal samples from humans and animals provided evidence for potential zoonotic transmission. In this study site, livestock may be a reservoir of human infection, especially in terms of assemblage AII and E. Our research revealed that the novel genotype belonging to assemblage AIII has been isolated only in buffalo and assemblage B appeared only in humans. Although there has been no conclusive evidence of zoonotic transmission of assemblage AIII and B, we assume that the unexpected human infection with all *Giardia* assemblages may be attributed to environmental contamination in the villages by human and animal feces. Due to a lack of a sewage system at the study site, all feces, both animal and human, can easily contaminate water. In this case, not only zoonotic transmission, but also “reverse zoonotic transmission” (zooanthroponotic), can occur. In fact, humans are also considered to be the source of infection in this environment, further affecting livestock productivity. With this in mind, it is essential for preventive methods for *Giardia* spp. transmission to be adapted to protect not only humans, but also animals and the environment.

## 5 Conclusions

Assemblage AII and E were detected in both animal and human stool samples in this study site. Based on these results, we conclude that zoonotic transmission cannot be ruled out in this area. Moreover, the detection of assemblage E from human stool samples is likely not coincidental, despite this study being the first to report assemblage E in humans in Vietnam. Although humans in this study site were considered to be the victims of zoonotic transmission of *Giardia* spp., there remains a considerable dearth of knowledge regarding the risk of *Giardia* transmission from animals to humans. Another possible explanation for this discovery is that humans themselves may contribute to the transmission of all assemblages, including assemblage E, through environmental contamination in this study site. In addition to the ‘animal-to-human’ route, there potentially might be transmission routes from ‘humans-to-humans’ and/or ‘humans-to-animals’ in this study site. Further environmental studies are needed to determine the major contributor to environmental contamination in relation to *Giardia* transmission.

The following are the supplementary data related to this article.

Supplementary Fig. S1 Distribution of buffalo stool sampling sites and *Giardia*-positive sites. Supplementary Fig. S1

Supplementary Fig. S2 Distribution of cattle stool sampling sites and *Giardia*-positive sites. Supplementary Fig. S2

Supplementary Fig. S3 Distribution of pig stool sampling sites and *Giardia*-positive sites. Supplementary Fig. S3

Supplementary Fig. S4 Distribution of dog stool sampling sites and *Giardia*-positive sites. Supplementary Fig. S4

Supplementary Table S1 Accession number for gene sequences obtained from GenBank used for *Giardia* assemblage identification. Supplementary Table S1

Diversity and frequency of all sequence results including single-nucleotide polymorphisms of Giardia assemblage A isolate at the *bg* gene (partial sequence between positions 111 and 585) Image 1

## Contributions

HI, TS, TT, AT and TY conceived and designed this study. THN, VDT and TDP helped in planning the study in Vietnam. HI, TS, THN, TDP, NLY, HTD and AHQP collected the field data, and HI, HTD, and AHQP conducted the laboratory work. HI drafted the manuscript. All authors have read and approved the final manuscript.

## Declaration of Competing Interest

All authors declare that they have no competing interests.
