# Supplementary material for: Molecular epidemiology of Giardia spp. in northern Vietnam: Potential transmission between animals and humans
Source: Parasite Epidemiol Control. 2020 Dec 24;12:e00193. doi: 10.1016/j.parepi.2020.e00193 (PMC7806796; doi:10.1016/j.parepi.2020.e00193)
Supplement: Supplementary Fig. S4 — Distribution of dog stool sampling sites and Giardia-positive sites. [file mmc4.docx]

**Supplemental Figure S4.** Distribution of dog stool sampling sites and *Giardia*-positive sites.

“GPS_No” are shown in the map.
