# Supplementary material for: Molecular epidemiology of Giardia spp. in northern Vietnam: Potential transmission between animals and humans
Source: Parasite Epidemiol Control. 2020 Dec 24;12:e00193. doi: 10.1016/j.parepi.2020.e00193 (PMC7806796; doi:10.1016/j.parepi.2020.e00193)
Supplement: Supplementary Table S1 — Accession number for gene sequences obtained from GenBank used for Giardia assemblage identification. [file mmc5.docx]

| Genotype | | Gene | | |
| --- | --- | --- | --- | --- |
| Assemblages | Sub-assemblage | *bg* | *gdh* | *tpi* |
| A | AI |  |  |  |
|  | AII | AY072723 (A2) | AB195223.1 (A2) | AY368157.1 |
|  |  | AY072724 (A3) |  |  |
|  | AIII | DQ650649 (A6) | DQ100288.1 | DQ650648 |
| B | BIII | AY072727 | AF069059 | AY228628.1 |
|  | BIV | AY072728 | AB295651 | KF922912.1 |
|  |  |  |  | L02116.1 |
|  |  |  | AY178749.1 |  |
| C |  | AY545646 | EF507635.1 | AY228641.1 |
|  |  |  | EF507631.1 |  |
| D |  | AY545647 | EF507629.1 | DQ246216.1 |
|  |  | AY545648 | EF507634.1 | DQ220289.2 |
| E |  | AY072729 (E1) | AB182127 | AY655705 (EII) |
|  |  | AY653159 (E3) | AY178741 |  |
|  |  |  |  | KJ668136 |
| F |  | AY647264 | EF507596.1 | KM870513 |
|  |  |  | EF507597.1 |  |
| G |  |  | AY178745.1 | MF671913.1 (GII) |
|  |  |  | AF069058.1 |  |

**Supplemental Table S1**. Accession number for gene sequences obtained from GenBank used for *Giardia* assemblage identification
