# Supplementary material for: Molecular epidemiology of Giardia spp. in northern Vietnam: Potential transmission between animals and humans
Source: Parasite Epidemiol Control. 2020 Dec 24;12:e00193. doi: 10.1016/j.parepi.2020.e00193 (PMC7806796; doi:10.1016/j.parepi.2020.e00193)
Supplement: Supplementary file 6 — Diversity and frequency of all sequence results including single-nucleotide polymorphisms of Giardia assemblage A isolate at the bg gene (partial sequence between positions 111 and 585) [file mmc6.docx]

**Supplemental_Table S2**:

Diversity and frequency of all sequence results including single-nucleotide polymorphisms of Giardia assemblage A isolate at the *bg* gene (partial sequence between positions 111 and 585)

| Sample ID (GenBank Acc. No.) | sample | Sub-assemblage | Nucleotide at position of reference sequence AY072724.1_AII_suptype-A3 | | | | | | | | | | | | | | | | | | | | | | | | | | | | | | | |
| --- | --- | --- | --- | --- | --- | --- | --- | --- | --- | --- | --- | --- | --- | --- | --- | --- | --- | --- | --- | --- | --- | --- | --- | --- | --- | --- | --- | --- | --- | --- | --- | --- | --- | --- |
|  |  |  | 113 | 132 | 149 | 176 | 243 | 249 | 287 | 293 | 295 | 325 | 348 | 356 | 362 | 372 | 415 | 418 | 426 | 438 | 448 | 455 | 456 | 467 | 495 | 528 | 529 | 556 | 558 | 561 | 568 | 572 | 576 | 583 |
|  |  |  | G | C | A | T | C | C | C | A | T | A | T | A | T | T | T | A | C | T | A | A | A | T | A | G | A | G | T | T | A | C | A | A |
| Ani_23 | Pig | AII | . | . | . | . | . |  | . | . | . | . |  | . | . |  |  | . | . | . | . | . |  | . | . |  | . | . |  |  | . | . | . | . |
| Ani_48 | Cattle | AII | . | . | . | . |  |  | . | . | . | . |  | . | . |  |  | . | . | . | . | . |  | . | . |  | . | . |  |  | . | . | . | . |
| 179_2, 1172_4 | Human^N^ | AII | . | . | . | . |  |  | . | . | . | . |  | . | . |  |  | . | . | . | . | . |  | . | . |  | . | . |  |  | . | . | . | . |
| CDS_659, CDS_1205, CDS_1392, CDS_1447, CDS_1469, CDS_1484, CDS_1534, CDS_1739 | Human^D^ | AII | . | . | . | . |  |  | . | . | . | . |  | . | . |  |  | . | . | . | . | . |  | . | . |  | . | . |  |  | . | . | . | . |
| Ani_23 (LC503600*) | Pig | AII | . | . | . | . |  |  | . | . | . | . |  | . | . |  |  | . | . | C | . | G |  | . | . |  | . | . |  |  | . | . | . | . |
| Ani_42 (LC503599*) | Pig | AII | . | . | . | . |  |  | . | G | . | . |  | . | G |  |  | . | . | . | . | . |  | . | . |  | . | . |  |  | . | . | . | . |
| CDS_1272 (LC503940*) | Human^D^ | AII | . | . | G | . |  |  | . | . | . | . |  | . | . |  |  | . | . | . | . | . |  | . | . |  | . | . |  |  | . | . | . | . |
| CDS_1285 (LC503941*) | Human^D^ | AII | . | . | . | . |  |  | . | . | C | . |  | G | . |  |  | . | . | . | . | . |  | . | . |  | . | . |  |  | . | . | . | . |
| CDS_1285 | Human^D^ | AII | . | R | . | C |  |  | . | . | . | . |  | . | . |  |  | . | . | . | . | . |  | . | . |  | . | . |  |  | . | . | . | . |
| CDS_1194 | Human^D^ | AII | . | . | . | . |  |  | . | . | . | . |  | . | . |  |  | . | . | . | . | . |  | . | . |  | . | . |  |  | R | . | R | . |
| CDS_1386 | Human^D^ | AII | . | . | . | . |  |  | . | . | . | . |  | . | . |  |  | . | . | . | . | . |  | . | G |  | R | . |  |  | . | . | . | . |
| CDS_1250 (LC503942*) | Human^D^ | AII | . | . | . | . |  |  | . | . | . | . |  | . | . |  |  | . | T | . | . | . |  | . | . |  | . | . |  |  | . | . | . | . |
| CDS_1679 (LC503943*) | Human^D^ | AII | . | . | . | . |  |  | . | . | . | G |  | . | . |  |  | . | . | . | G | . |  | . | . |  | . | . |  |  | G | . | . | . |
| CDS_1484 (LC503944*) | Human^D^ | AII | . | . | . | . |  |  | . | . | C | . |  | . | . |  |  | . | . | . | . | . |  | . | . |  | . | . |  |  | . | . | . | . |
| CDS_763 (LC503945*) | Human^D^ | AII | . | . | . | . |  |  | . | . | . | . |  | . | . |  |  | G | . | . | . | . |  | . | . |  | . | A |  |  | . | . | . | . |
| CDS_1679 (LC503946*) | Human^D^ | AII | A | . | . | . |  |  | T | . | . | . |  | . | . |  |  | . | . | . | . | . |  | C | . |  | . | . |  |  | . | T | . | G |
| Ani_55, Ani_56 (LC503598*) | Buffalo | AIII |  |  |  |  | T | T |  |  |  |  | C |  |  | C | C |  |  |  |  |  | G |  |  | A |  |  | C | C |  |  | G |  |
| DQ650649.1 | . | AIII |  |  |  |  |  | T |  |  |  |  | C |  |  | C | C |  |  |  |  |  | G |  |  | A |  |  | C | C |  |  | G |  |

^N^: non-diarrheal sample, ^D^: diarrheal sample, * The novel sequences without heterogeneous positions were newly submitted to GenBank

**Supplemental_Table S3**:

Diversity and frequency of all sequence results including single-nucleotide polymorphisms of Giardia assemblage B isolate at the *bg* gene (partial sequence between positions 111 and 585)

| Sample ID (GenBank Acc. No.) | Sample | Nucleotide at position of reference sequence AY072727.1_BIII | | | | | | | | | | | | | | | | | | | | | | | | | | | |
| --- | --- | --- | --- | --- | --- | --- | --- | --- | --- | --- | --- | --- | --- | --- | --- | --- | --- | --- | --- | --- | --- | --- | --- | --- | --- | --- | --- | --- | --- |
|  |  | 129 | 132 | 135 | 159 | 165 | 183 | 185 | 219 | 227 | 240 | 276 | 288 | 309 | 327 | 339 | 348 | 387 | 390 | 414 | 415 | 423 | 438 | 450 | 457 | 504 | 535 | 551 | 576 |
|  |  | G | T | G | G | C | A | A | C | A | T | C | C | C | G | C | C | A | C | C | C | C | C | C | A | C | A | G | G |
| 63_5, 63_7 | Human^N^ | . | . | . | . | . | . | . | . | . | . | . | . | . | . | . | . | . | . | . | . | . | . | . | . | . | . | . | . |
| CDS_1298, CDS_1368, CDS_1386, CDS_1392, CDS_1469, CDS_1696 | Human^D^ | . | . | . | . | . | . | . | . | . | . | . | . | . | . | . | . | . | . | . | . | . | . | . | . | . | . | . | . |
| CDS_1234, CDS_1236, CDS_1274, CDS_1308, CDS_1297, CDS_1921 (LC503947*) | Human^D^ | . | . | . | . | . | . | . | . | . | . | . | . | . | . | . | . | . | . | . | . | T | . | T | . | . | . | . | . |
| CDS_1297, CDS_1298 | Human^D^ | . | . | . | . | . | . | . | . | . | . | . | . | . | . | . | . | . | . | . | . | Y | . | Y | . | . | . | . | . |
| CDS_1234 (LC503948*) | Human^D^ | . | . | . | . | . | . | . | . | . | . | . | . | . | . | . | . | . | . | . | . | . | . | . | . | . | G | . | . |
| CDS_1534 | Human^D^ | . | . | . | . | . | . | . | . | . | . | . | . | . | . | . | . | . | . | . | . | . | . | . | . | . | . | . | R |
| CDS_1697 | Human^D^ | . | . | . | . | . | . | . | . | . | . | . | . | . | . | . | . | . | . | . | . | . | . | . | . | . | . | R | . |
| CDS_992 (LC503950*) | Human^D^ | . | . | A | . | . | G | . | . | . | . | . | . | . | . | . | . | . | . | . | . | . | . | . | . | . | . | . | . |
| CDS_1447 | Human^D^ | R | . | . | . | . | . | . | . | R | . | . | . | . | . | . | . | . | . | . | . | . | . | . | R | . | . | . | . |
| CDS_256 | Human^D^ | . | Y | . | Y | . | G | . | . | . | Y | . | Y | T | A | Y | Y | . | Y | . | . | . | . | . | . | . | . | . | . |
| CDS_257 | Human^D^ | . | . | . | . | Y | . | . | . | . | . | . | . | Y | . | . | . | . | . | . | . | . | . | . | . | Y | . | . | . |
| CDS_258 | Human^D^ | . | . | . | . | Y | R | . | Y | . | . | . | . | Y | . | . | . | . | . | . | . | . | . | . | . | Y | . | . | . |
| CDS_261 | Human^D^ | . | . | . | . | Y | . | . | . | . | . | . | . | T | . | . | . | . | . | . | . | . | . | . | . | . | . | . | . |
| CDS_1192 (LC503951*) | Human^D^ | . | . | . | . | . | . | . | . | . | . | T | . | T | . | . | . | . | . | T | . | . | . | . | . | . | . | . | . |
| CDS_1250 | Human^D^ | . | . | . | . | . | . | . | . | . | . | . | Y | . | . | . | . | R | . | . | Y | T | Y | T | . | . | . | R | R |

^N^: non-diarrheal sample, ^D^: diarrheal sample, * The novel sequences without heterogeneous positions were newly submitted to GenBank

**Supplemental_Table S4**: Diversity and frequency of all sequence results including single-nucleotide polymorphisms of Giardia assemblage E isolate at the *bg* gene (partial sequence between positions 18 and 492)

| Sample ID (GenBank accession no.) | Sample | sub-type | Nucleotide at position of reference sequence AY653159.1_E-3 | | | | | | | | |
| --- | --- | --- | --- | --- | --- | --- | --- | --- | --- | --- | --- |
|  |  |  | 130 | 160 | 162 | 213 | 245 | 253 | 275 | 379 | 479 |
|  |  |  | G | A | A | A | T | T | T | G | C |
| Ani_11, Ani_32, Ani_33, Ani_40, Ani_58, Ani_90, Ani_114, Ani_146, Ani_147 | Cattle | E3 | . | . | . | . | . | . | . | . | . |
| Ani_6, Ani_14, Ani_19, Ani_23, Ani_47 | Pig | E3 | . | . | . | . | . | . | . | . | . |
| Ani_7, Ani_140 | Dog | E3 | . | . | . | . | . | . | . | . | . |
| Ani_15 | Buffalo | E3 | . | . | . | . | . | . | . | . | . |
| 63_5, 63_6, 800_2, 1160_6, 1245_7 | Human non-diarrheal | E3 | . | . | . | . | . | . | . | . | . |
| CDS_256, CDS_259, CDS_658, CDS_659, CDS_763 | Human diarrheal | E3 | . | . | . | . | . | . | . | . | . |
| Ani_23 | Pig | E3 | . | . | R | R | . | T | . | G | . |
| Ani_23 | Pig | E3 | . | . | G | . | . | T | . | G | . |
| CDS_256 (LC503938*) | Human diarrheal | E3 | A | . | . | . | . | . | C | . | . |
| CDS_660 (LC503939*) | Human diarrheal | E3 | . | . | . | . | C | . | . | . | . |
| Ani_73 | Pig | E1 | . | . | . | . | . | C | . | A | . |
| AY072729.1 | . | E1 |  |  |  |  |  | C |  | A |  |

**Supplemental_Table S5**:

Diversity and frequency of all sequence results including single-nucleotide polymorphisms of Giardia assemblage A isolate at the *gdh* gene (partial sequence between positions 48 and 440)

| Sample ID (GenBank Acc. No.) | Sample | Sub-assemblage | Nucleotide at position of reference sequence AB195223.1 | | | | | | | | | | | | | | | | | |
| --- | --- | --- | --- | --- | --- | --- | --- | --- | --- | --- | --- | --- | --- | --- | --- | --- | --- | --- | --- | --- |
|  |  |  | 48 | 60 | 99 | 162 | 180 | 186 | 218 | 312 | 315 | 321 | 324 | 344 | 360 | 373 | 409 | 411 | 423 | 426 |
|  |  |  | C | T | C | C | T | A | T | G | C | C | A | A | C | A | G | T | C | T |
| 179_2, 1172_4 | Human non-diarrheal | AII |  |  |  |  |  |  |  |  |  |  |  |  |  |  |  |  |  |  |
| CDS_1234 | Human diarrheal | AII |  |  |  |  |  |  |  |  |  |  |  |  |  |  |  |  |  |  |
| CDS_1250 (LC503952 )* | Human diarrheal | AII |  |  |  |  |  |  |  |  |  |  |  | G |  |  |  |  |  |  |
| CDS_1272 (LC503953 )* | Human diarrheal | AII |  |  |  |  |  |  |  |  |  |  |  |  |  | G | A |  |  |  |
| CDS_1368 | Human diarrheal | AII |  |  |  |  |  |  | C |  |  |  |  |  |  |  |  |  |  |  |
| AB808753.1 | . | AII |  |  |  |  |  |  | C |  |  |  |  |  |  |  |  |  |  |  |
| Ani_55, Ani_56 | Buffalo | AIII | G | C | T | A | C | G |  | C | T | T | G |  | T |  |  | C | T | C |
| DQ100288.1 | . | AIII | G | C | T | A | C | G |  | C | T | T | G |  | T |  |  | C | T | C |

**Supplemental_Table S6**: Diversity and frequency of all sequence results including single-nucleotide polymorphisms of *G.duodenalis* assemblage B isolate at the *gdh* gene (partial sequence between positions 1 and 393)

| Sample ID (GenBank Acc. No.) | Sample | Nucleotide at position of reference sequence AB295651.1_BIV | | | | | | | | | | | | | | | | | | |
| --- | --- | --- | --- | --- | --- | --- | --- | --- | --- | --- | --- | --- | --- | --- | --- | --- | --- | --- | --- | --- |
|  |  | 16 | 52 | 100 | 172 | 175 | 179 | 190 | 191 | 211 | 250 | 265 | 283 | 304 | 307 | 332 | 349 | 355 | 370 | 372 |
|  |  | T | T | C | C | C | T | C | G | G | C | T | T | C | G | A | C | A | G | A |
| 63-7, 800-2, 1160-6, 1245-7 | Human^N^ |  |  |  |  |  |  |  |  |  |  |  |  |  |  |  |  |  |  |  |
| CDS_1192, CDS_1392 | Human^D^ |  |  |  |  |  |  |  |  |  |  |  |  |  |  |  |  |  |  |  |
| CDS_1484 | Human^D^ | Y |  | Y |  |  | K |  | R |  |  |  |  |  |  |  |  |  |  |  |
| CDS_1205, CDS_1368, CDS_1274, CDS_1285, CDS_1392, CDS_1534, CDS_1660 (AB569386.1) | Human^D^ |  |  | T |  |  |  |  |  |  |  |  |  |  |  |  |  |  |  |  |
| CDS_1234 (LC503957) * | Human^D^ |  |  | T |  |  |  |  |  |  |  | C |  |  |  |  |  |  |  |  |
| CDS_1386 (AB618784.1) | Human^D^ |  |  | T |  |  |  |  |  |  |  |  |  | T |  |  |  |  |  |  |
| CDS_1297 | Human^D^ |  |  | T |  |  | K |  |  |  |  |  |  |  |  |  |  |  |  |  |
| CDS_1298 | Human^D^ |  |  | T |  |  | K |  |  |  |  |  |  | Y |  |  |  |  |  |  |
| CDS_1447 | Human^D^ |  |  | Y |  |  | G |  |  |  |  |  |  | Y |  |  |  |  |  |  |
| CDS_1696 | Human^D^ |  |  | Y |  |  | K |  |  |  |  |  |  | Y |  |  |  |  |  |  |
| CDS_256 | Human^D^ |  |  |  |  | Y |  |  |  | R |  |  | Y | Y |  |  |  | R |  |  |
| CDS_257 | Human^D^ |  |  |  |  | Y |  |  |  | R |  |  | Y |  |  |  |  | R |  |  |
| CDS_1469 (LC503962) * | Human^D^ |  |  |  |  |  | G |  |  |  |  |  |  |  |  |  |  |  |  |  |
| CDS_1236, CDS_1272, CDS_1447, CDS_1534, CDS_1697, CDS_1698 (LC503958) * | Human^D^ |  |  |  |  |  | G |  |  |  |  |  |  | T |  |  |  |  |  |  |
| CDS_257 (KT124839.1) | Human^D^ |  |  |  |  |  |  |  |  | A |  |  | C |  |  |  |  | G |  |  |
| CDS_258 (LC430569.1) | Human^D^ |  |  |  |  |  |  |  |  | A |  |  | C |  |  |  |  |  |  |  |
| CDS_259_NIHE0300 | Human^D^ |  |  |  |  |  |  |  |  | A |  |  | Y |  |  |  |  |  |  |  |
| CDS_259_NIHE2474, DEC_261_NIHE2445 | Human^D^ |  |  |  |  |  |  |  |  | R |  |  | Y | Y |  |  |  | R |  |  |
| CDS_259 (LC503954) * | Human^D^ |  |  |  |  |  |  |  |  | A |  |  | C |  |  |  |  | G |  |  |
| CDS_259_NIHE2451 | Human^D^ |  |  |  |  |  |  |  |  | R |  |  | Y |  |  |  |  | R |  |  |
| CDS_261 (LC503955) * | Human^D^ |  |  |  |  |  |  |  |  | A |  |  | C |  |  |  |  |  |  |  |
| CDS_658 (LC503956) * | Human^D^ |  |  |  |  |  |  |  |  | A |  |  | C |  | A |  |  | G |  |  |
| CDS_659 (KT124839.1) | Human^D^ |  |  |  |  |  |  |  |  | A |  |  | C |  |  |  |  | G |  |  |
| 63-6, 63-7 (LC504285) * | Human^N^ |  |  |  |  |  |  |  |  |  | T |  |  | T |  |  | T |  | A |  |
| 63-5, 63-7 | Human^N^ |  |  |  |  |  |  |  |  |  | Y |  |  | Y |  |  | Y |  | R |  |
| CDS_256, CDS_257 (LC430569.1) | Human^D^ |  |  |  |  |  |  |  |  |  |  |  | C |  |  |  |  |  |  |  |
| CDS_992 | Human^D^ |  |  |  |  |  |  |  |  |  |  |  | Y |  |  |  |  |  |  |  |
| 800-2 (AY178749) | Human^N^ |  |  |  |  |  |  |  |  |  |  |  |  | T |  |  |  |  |  |  |
| CDS_658, CDS_1484 (AY178749) | Human^D^ |  |  |  |  |  |  |  |  |  |  |  |  | T |  |  |  |  |  |  |
| CDS_1308 (LC503960) * | Human^D^ |  |  |  |  |  |  |  |  |  |  |  |  |  |  | G |  |  |  | G |
| CDS_1308 | Human^N^ |  |  |  |  |  |  |  |  |  |  |  |  |  |  |  |  |  | A |  |
| AF069059.1_BIII | . |  | C | T | T |  | T | T |  |  |  |  | C |  |  |  |  | G |  |  |

^N^: non-diarrheal sample, ^D^: diarrheal sample, * The novel sequences without heterogeneous positions were newly submitted to GenBank

**Supplemental_Table S7**:

Diversity and frequency of all sequence results including single-nucleotide polymorphisms of Giardia assemblage E isolate at the *gdh* gene (partial sequence between positions 48 and 440)

| Sample ID (GenBank Acc. No.) | Sample | Nucleotide at position of reference sequence AB182127.1 | | | | | | | | | |
| --- | --- | --- | --- | --- | --- | --- | --- | --- | --- | --- | --- |
|  |  | 149 | 153 | 188 | 225 | 229 | 238 | 255 | 336 | 362 | 372 |
|  |  | C | G | A | G | T | G | T | T | T | A |
| Abu_11, Ani_32, Ani_33, Ani_40, Ani_58, Ani_90, Ani_114, Ani_146, Ani_147 | Cattle |  |  |  |  |  |  |  |  |  |  |
| Ani_7, Ani_140 | Dog |  |  |  |  |  |  |  |  |  |  |
| Ani_14, Ani_23 | Pig |  |  |  |  |  |  |  |  |  |  |
| 63-5, 63-6, 1245-7 | Human non-diarrheal |  |  |  |  |  |  |  |  |  |  |
| CDS_659 | Human diarrheal |  |  |  |  |  |  |  |  |  |  |
| Ani_6 (LC504283*) | Pig | T |  |  |  |  |  | C |  |  |  |
| Ani_80 (LC504281*) | Cattle |  | T |  |  |  |  |  |  |  |  |
| Ani_47 (LC504282*) | Pig |  |  |  | A |  |  |  |  |  |  |
| Ani_48 (LC504280*) | Cattle |  |  |  |  |  | A |  |  |  |  |
| 1160-6 (LC504287*) | Human non-diarrheal |  |  |  |  | C |  |  |  | C |  |
| Ani_19 (LC504284*) | Pig |  |  |  |  |  |  |  |  | A |  |
| CDS_660 (LC503966*) | Human diarrheal |  |  | G |  |  |  |  |  |  |  |
| Ani_73 | Pig |  |  |  |  |  |  |  | C |  | G |
| AY178741.1 | . |  |  |  |  |  |  |  | C |  | G |

**Suppleimental_Table S8**: Diversity and frequency of all sequence results including single-nucleotide polymorphisms of Giardia assemblage A at the *tpi* gene (partial sequence between 22 to 511)

| Sample ID (Gen Bank Acc. No.) | Sample | Sub-assemblage | Nucleotide at position of reference sequence AY368157.1_AII | | | | | | | | | | | | | | | | | | |
| --- | --- | --- | --- | --- | --- | --- | --- | --- | --- | --- | --- | --- | --- | --- | --- | --- | --- | --- | --- | --- | --- |
|  |  |  | 22 | 79 | 94 | 97 | 103 | 106 | 115 | 119 | 130 | 148 | 160 | 175 | 217 | 283 | 338 | 380 | 385 | 478 | 484 |
|  |  |  | C | T | C | G | C | C | C | G | G | A | A | A | C | A | C | T | T | G | C |
| 179_2, 1172_4 | Human non-diarrheal | AII |  |  |  |  |  |  |  |  |  |  |  |  |  |  |  |  |  |  |  |
| Ani_55, Ani_56 (LC503768*) | Buffalo | AIII | T | C | T |  | T | T | T | T | A | G | G | G | T | G | T | C | C | T | T |
| DQ650648.1 | . | AIII | C | C | T | A | T | T | T | T | A | G | G | G | T | A | T | C | C | T | T |

**Supplemental_Table S9**: Diversity and frequency of all sequence results including single-nucleotide polymorphisms of Giardia B at the *tpi* gene (partial sequence between 22 to 511)

| Sample ID (GenBank Acc. No.) | Sample | Nucleotide at position of reference sequence AY228628.1 | | | | | | | | | | | | | | | | | | |
| --- | --- | --- | --- | --- | --- | --- | --- | --- | --- | --- | --- | --- | --- | --- | --- | --- | --- | --- | --- | --- |
|  |  | 25 | 31 | 77 | 151 | 154 | 175 | 196 | 249 | 257 | 266 | 283 | 298 | 319 | 388 | 424 | 457 | 469 | 479 | 496 |
|  |  | G | T | C | C | C | A | G | G | C | A | A | C | T | A | T | A | A | G | G |
| 63-7, 63-5 | human_non-diarrheal |  |  |  |  |  |  |  |  |  |  |  |  |  |  |  |  |  |  |  |
| 63-5, 63-7 (LC503771) * | human_non-diarrheal |  | C |  | T | T | G | A |  |  |  |  |  |  |  |  |  |  |  | A |
| KF922912.1_BIV |  | A |  |  | T | T |  | A |  |  |  |  |  |  |  |  |  |  |  |  |
| 1160-2 | human_non-diarrheal | A |  |  | T | T |  | A |  |  |  |  |  |  |  |  |  |  |  |  |
| JX994248.1 |  | A | C | T | T | T |  |  |  | T | G | G |  |  | G |  | C | G |  | A |
| 1245-7 | human_non-diarrheal | A | C | T | T | T |  |  |  | T | G | G |  |  | G |  | C | G |  | A |
| CDS_1192 | human_diarrheal | A |  | T | T |  |  | R |  |  |  |  | Y |  |  |  |  |  |  |  |
| CDS_256, CDS_257, CDS_258, CDS_259, CDS_261 | human_diarrheal |  | C | T | T |  |  | A | R |  |  |  |  |  |  |  |  |  |  |  |
| CDS_261 | human_diarrheal |  | C |  | T |  |  |  |  | Y | R | G |  | C |  |  |  | G |  |  |
| CDS_1297, CDS_1298 (LC503968) * | human_diarrheal |  | C |  | T |  |  |  |  |  |  |  |  |  | G | C |  | G |  |  |
| CDS_1298 | human_diarrheal |  | C |  | Y |  |  |  |  |  |  |  |  |  | R | Y |  | G |  |  |
| CDS_1297 | human_diarrheal |  | C |  |  | Y |  |  |  |  |  |  |  |  | R | Y |  | G |  |  |
| CDS_1297 (LC503967) * | human_diarrheal |  | C |  |  |  |  |  |  |  |  |  |  |  | G | C |  | G |  |  |
| CDS_1298 | human_diarrheal |  | C |  |  |  |  |  |  |  |  |  |  |  | R | Y |  | G | C |  |

**Supplemental_Table S10**: Diversity and frequency of all sequence results including single-nucleotide polymorphisms of Giardia assemblage E at the *tpi* gene (partial sequence between 9 to 498)

| Sample ID (GenBank Acc. No.) | Sample | Nucleotide position from the start of the gene AY655705.1 | | | | | | |
| --- | --- | --- | --- | --- | --- | --- | --- | --- |
|  |  | 45 | 66 | 82 | 125 | 335 | 444 | 462 |
|  |  | T | T | G | C | A | A | A |
| Ani_40, Ani_58, Ani_90, Ani_114, Ani_146, Ani_147 | cattle |  |  |  |  |  |  |  |
| Ani_11 (LC503769*) | cattle |  |  |  | T |  |  |  |
| Ani_73 | pig | C | C | A |  | G | G | G |
| KJ668136 | . | C | C | A |  | G | G | G |
